# Pleiotropic genes linking congenital hypogonadotropic hypogonadism and cleft lip/palate: evidence from a genomic CHH cohort study

**DOI:** 10.1038/s41431-025-02005-6

**Published:** 2026-01-14

**Authors:** Fernanda de Azevedo Correa, Imen Habibi, Jing Zhai, Michela Adamo, Yi Wang, Alexia Boizot, Yassine Zouaghi, Anita Rauch, Sandra Pekic, Richard Quinton, Marco Bonomi, Biagio Cangiano, Waljit S. Dhillo, Christa E. Fluck, Attila Nemeth, Pierre-Marc Bouloux, Jean-Marc Ferrara, Duarte Pignatelli, Zita Halász, Cecilia Perdices-Lopez, Andrea Messina, Nicolas J. Niederländer, Federico Santoni, James S. Acierno, Nelly Pitteloud

**Affiliations:** 1 Department of Endocrinology, Diabetology, and Metabolism, Lausanne University Hospital, Lausanne, Switzerland; 2 Faculty of Biology and Medicine, University of Lausanne, Lausanne, Switzerland; 3 Institute of Medical Genetics, University of Zurich, Zurich, Switzerland; 4 Department of Neuroendocrinology, Clinic for Endocrinology, Diabetes and Diseases of Metabolism, Faculty of Medicine, University Clinical Center, University of Belgrade, Beograd, Serbia; 5 Department of Metabolism, Digestion & Reproduction, Imperial College London, London, UK; 6 Department of Medical Biotechnology and Translational Medicine, University of Milan, Milan, Italy; 7 Department of Endocrine and Metabolic Diseases, IRCCS Istituto Auxologico Italiano, Milan, Italy; 8 Section of Endocrinology and Investigative Medicine, Imperial College London, London, UK; 9 Division of Pediatric Endocrinology, Diabetology and Metabolism, Department of Pediatrics, Bern University Hospital, University of Bern, Bern, Switzerland; 10 Department of Biomedical Research, University of Bern, Bern, Switzerland; 11 Department of Endocrinology, St John’s Hospital, Budapest, Hungary; 12 Center for Neuroendocrinology, Royal Free Campus, University College Medical School, London, UK; 13 Rue du Curtil-Maillet Yverdon-les-Bains, Yverdon-les-Bains, Switzerland; 14 Department of Endocrinology, Hospital S João, Porto, Portugal; 15 Department of Biomedicine, Faculty of Medicine of the University of Porto, Porto, Portugal; 16 IPATIMUP Research Institute, Porto, Portugal; 17 Bókay Street Department, Paediatric Centre, Faculty of Medicine, Semmelweis University, Budapest, Hungary

**Keywords:** Endocrine reproductive disorders, Endocrine reproductive disorders, Genetics research, Disease genetics

## Abstract

Congenital hypogonadotropic hypogonadism (CHH) is a rare and genetically heterogeneous disorder characterized by absent or incomplete puberty due to impaired gonadotropin-releasing hormone (GnRH) function. A subset of individuals with CHH also present with developmental anomalies, including midline defects such as cleft lip and/or palate (CLP). This study investigates the genetic overlap between CHH and CLP. A total of 336 individuals diagnosed with CHH were clinically assessed for associated phenotypes, including CLP. High-throughput sequencing was performed using a targeted gene panel encompassing known CHH- and CLP-related genes. Variants were analyzed and classified according to the American College of Medical Genetics and Genomics (ACMG) criteria for pathogenicity. CLP was present in 21 patients with CHH (6%). Pathogenic or likely pathogenic variants in genes associated with both CHH and CLP—such as *FGFR1* and *CHD7*—were identified in eight individuals. Furthermore, 17% of the patients with CHH without CLP harbored deleterious variants in genes implicated in clefting, including *DVL3*, *PLCB4*, *NIPBL*, and *EDNRA*. Evidence of digenic inheritance involving both CHH- and CLP-related genes was observed in multiple cases. *FGFR1* variants were the most frequently detected and were commonly associated with anosmia and additional developmental anomalies. These findings highlight a genetic and phenotypic continuum between CHH and CLP, underscoring the involvement of shared developmental pathways. The high prevalence of *FGFR1* variants in patients with CHH and CLP supports its role as a pleiotropic gene. Understanding the overlapping genetic mechanisms may enhance diagnostic precision and inform personalized management strategies for affected individuals.

## Introduction

Congenital hypogonadotropic hypogonadism (CHH) is a rare heterogeneous genetic disorder occurring in 1 in 10,000 births [1]. It is characterized by the absence of puberty and subsequent infertility due to gonadotropin-releasing hormone (GnRH) deficiency [2]. The developmental link between the GnRH and olfactory systems accounts for the frequent association of CHH with anosmia, a condition known as Kallmann syndrome (KS) [3]. Over the past 30 years, genetic studies in both humans and mice have identified more than 40 loci harboring rare, high-impact variants implicated in CHH pathogenesis [4, 5]. Pathogenic (P) or likely pathogenic (LP) variants in known CHH genes are found in around 50% of patients, highlighting the need to identify additional genetic contributors [6]. Oligogenic inheritance—mutations in two or more genes—likely contributes to the variable expressivity seen both within and between affected families carrying the same mutation. It is presently observed in approximately 15% of patients, this landscape is likely to increase as more genes are discovered [7].

Patients with CHH often present with additional developmental anomalies, most commonly anosmia in around 50% of cases, but also including cleft lip and/or palate (CLP) [5, 8]. Our previous work has shown phenotypic and genetic overlap between CHH and several other syndromic conditions, such as septo-optic dysplasia (SOD), combined pituitary hormone deficiency (CPHD), and CHARGE syndrome (characterized by coloboma, heart defects, choanal atresia, growth retardation, genital anomalies, and ear abnormalities) [9, 10].

CLP is among the most common congenital malformations, with a complex etiology and substantial impact on morbidity and quality of life. It affects approximately 1 in 700 live births, with prevalence varying significantly by ethnicity [11]. Based on anatomical, embryological, and genetic evidence, orofacial clefts are typically classified into two major categories: cleft lip with or without cleft palate (CLP) and cleft palate only (CP). The clinical burden of these anomalies depends on severity and may include feeding difficulties, speech impairments, ear, nose and throat complications, dental and orthodontic issues, aesthetic concerns, and psychosocial challenges that can persist into adulthood [11]. Clefts may occur as part of a broader syndromic presentation involving additional developmental anomalies or, in isolation, referred to as non-syndromic CLP. Approximately 70% of CLP and 50% of CP cases are classified as non-syndromic [12]. The etiology of CLP is multifactorial, involving both genetic and environmental determinants. The genetic architecture is complex, with mutations in several genes already been described. Genome-wide association studies also contributed in the dentification of several genes and variants that are associated with CLP susceptibility [11]. Five to ten percent of probands with CHH also exhibit CLP [8, 13–15]. This prevalence increases to 30–40% among patients with CHH carrying *FGFR1* mutations, highlighting a notable clinical and genetic overlap between CHH and CLP [8, 16]. Moreover, *FGFR1* mutations have also been implicated in isolated cases of CLP [17]. While prior studies have evaluated the role of CHH-associated genes—such as *FGFR1* and *FGF8*—in cohorts with CLP [18], the reverse approach, namely, the investigation of CLP-related genes in patients with CHH, has not been explored.

The aim of this study was to expand our understanding of the clinical and genetic overlap between CHH and CLP. Through high-throughput sequencing and comprehensive variant analysis, we sought to identify shared molecular mechanisms, uncover novel CLP-related genes implicated in CHH, investigate potential digenic inheritance patterns, and gain deeper insight into the developmental pathways connecting these two conditions.

## Material and methods

### Patients and clinical evaluation

The CHH cohort was comprised 336 unselected probands. The patients are part of Prof. N. Pitteloud’s cohort that she has assembled over more than 2 decades. This cohort comprises samples from patients coming from several medical centers all over the world, yet mainly from Europe. All participants provided written informed consent, and the study was conducted in accordance with the Declaration of Helsinki. Diagnosis of CHH was established based on the criteria outlined in the European Consensus Statement on CHH [19]. Specifically, CHH was defined by: (i) absent or incomplete puberty by age 17; (ii) low or inappropriately normal gonadotropin levels in the context of low serum sex steroids (testosterone in males or estradiol in females); (iii) normal hypothalamic and pituitary anatomy on imaging; (iv) otherwise normal anterior pituitary function, and (v) absence of iron overload.

Olfactory function was assessed through self-report and/or formal testing using the University of Pennsylvania Smell Identification Test [16]. All participants underwent a comprehensive medical examination and review of clinical history. Magnetic resonance imaging was used to evaluate the olfactory bulbs and tracts, as well as the hypothalamic-pituitary region. Diagnosis of CLP was based on clinical evaluation, supplemented by radiographs and computed tomography imaging when necessary.

## Genetic analysis

### DNA extraction and sequencing

Genomic DNA was extracted from blood samples of the 336 patients with CHH and their available relatives using standard protocols provided by the manufacturer. WES and WGS were performed using the Agilent SureSelect v5 exome capture kit and the DNBSEQ-T20×2 platform, respectively. Sequencing was carried out on the Illumina HiSeq 2500 platform, following previously described protocols [7].

The resulting raw sequencing data (FASTQ files) were processed using an in-house bioinformatics pipeline built around the Sentieon DNASeq toolkit, a GATK-compliant platform [20, 21]. Reads were aligned to the human reference genome (GRCh38), and single-nucleotide variants (SNVs) as well as small insertions and deletions (Indels <50 bp) were identified. Variant annotation included population allele frequencies from gnomAD v4.1 [22] and in silico predictions of pathogenicity using tools such as CADD [23], SpliceAI [24], REVEL [25] and Alphamissense [26] facilitated by ANNOVAR [27]. Sequencing was performed to a minimum average coverage depth of 30× per sample.

Annotated variants were subsequently visualized, filtered, and prioritized using GenMasterTable, a variant interpretation platform developed in-house [28].

#### SNVs analysis

Variants were selected based on one or more of the following criteria: (i) predicted loss-of-function variants, including nonsense (stop-gain), frameshift, and canonical splice site mutations (± 2 bp from an exon); (ii) missense variants; (iii) in-frame insertions or deletions; or (iv) a SpliceAI-predicted probability > 0.8 of causing a splicing defect [24]. All variants met GATK quality control thresholds, including a minimum quality score of 50.

Filtered variants were assessed against two panels: panel A—a curated CLP gene panel, which includes 162 genes and panel B—a CHH-specific gene panel with 41 genes (Supplementary Table 1). There are two overlapping genes in both panels (A + B), *FGFR1* and *CHD7*, since they have already been associated with both phenotypes. Panel A was assembled using PanelApp, a publicly accessible, expert-curated virtual gene panel resource [29], downloaded on April 20, 2023. Panel B comprises 41 genes known to be associated with CHH as documented in the OMIM database (Supplementary Table 1).

Consistent with inheritance patterns commonly observed in rare diseases, variants were considered potentially pathogenic according to the following thresholds: (i) autosomal recessive, MAF < 1% (ii) autosomal dominant MAF < 0.1% and X-linked MAF < 0.1%. Variants were further annotated and classified for pathogenicity using VarSome [30], based on the guidelines of the American College of Medical Genetics and Genomics [31]. A variant was considered novel if it had not been previously reported in the medical literature or was absent from the human gene mutation database [32] and ClinVar https://www.ncbi.nlm.nih.gov/clinvar/. All prioritized variants were confirmed by Sanger sequencing, using previously published protocols [33], and, when possible, familial segregation analysis was performed.

## Results

### Probands with CHH and CLP

Among the 336 patients with CHH, 21 (6%) presented with CLP. Heterozygous P or LP variants in CLP-related genes were identified in 10 of these individuals (5 males; Table 1); among these 10 individuals, eight harbored variants in genes known to be implicated in both CLP and CHH individually. Of note, *FGFR1* and *CHD7* are both CLP and CHH-related genes and are listed in panels A and B (Supplementary Table S1).

**Table 1:** Genetic and clinical data of patients with CHH and cleft lip and/or palate.

Patient	Sex	Genes	Nucleotide change	Amino acid change	Inheritance	ACMG class	Olfaction	Neuroimaging	Associated phenotypes
1	M	*FGFR1*	c.290 G > T	p.Gly97Val	Paternal	P	Anosmia	Normal pituitary	None
2	M	*FGFR1*	c.1905C > G	p.Asn635Lys	Maternal	P	Anosmia	NA	None
3	F	*FGFR1*	c.103delGinsCT	p.Gly35Leufs*15	Maternal	LP	Anosmia	NA	None
4	M	*FGFR1*	c.752 C > A	p.Thr251Lys	Paternal	LP	Normal	Left OB aplasia, right OB hypoplasia normal pituitary	Growth retardation, synkinesia, dental agenesis, myasthenia gravis
5	F	*FGFR1*	c.828_828delinsAGG	p.Pro366Glyf*4	Maternal	LP	Anosmia	Left OB aplasia, dysgenesis of corpus callosum, bilateral hypoplasia of optic nerve	Hearing loss, ID/DD, SOD, high-arched palate
6	M	*FGFR1*	c.1546 + 1 G > A^a^	–	Maternal	LP	Normal	NA	None
7	F	*FGFR1*	c.779 G > T	p.Gly260Val	Unknown	LP	Anosmia	Normal pituitary	Ocular motor dyspraxia in childhood; bifid nose; agenesis of left upper canine and ectopic eruption of other teeth
8	F	*CHD7*	c.3320 C > T	p.Ala1107Val	Maternal	LP	Anosmia	Normal OB, non-functioning microadenoma 0.6 mm	Hearing loss, synkinesia, scoliosis, mitral valve prolapse, AVNRT, osteoporosis, hip dysplasia, elbow valgus, astigmatism, choroidal nevus, VUR, unilateral renal hypoplasia
9	F	*SOX9*	c.509 C > A	p.Pro170Arg	de novo	P	Anosmia	Absence of the OB	Hearing loss, growth retardation, synkinesia, ear anomalies, ID/DD, bicuspid aortic valves, strabismus, metatarsus varus, brachydactyly, low-set ears
10	M	*CTNND1*	c.2809-1 G > C^a^	–	Paternal	LP	Anosmia	NA	Hearing loss, hypertelorism, double permanent teeth, cryptorchidism (left) brachydactyly

All variants were in heterozygous state. All genes depicted at this table have autosomal dominant (AD) mode of inheritance (OMIM).

*M* male; *F* female; *ACMG* American College of Medical Genetics; *P* pathogenic; *LP* likely pathogenic; *NA* not available; *OB* olfactory bulb; *ID/DD* intellectual deficiency/developmental delay; *SOD* septo-optic dysplasia; *AVNRT* atrioventricular nodal re-entry tachycardia; *VUR* vesicoureteral reflux.

^a^Predicted to be splice-altering using the Splice AI tool.

Among the patients with CHH and CLP, the most frequently observed deleterious variants were in *FGFR1* (*n* = 7). Among these, five patients exhibited anosmia or hyposmia, and three had complex associated phenotypes: patient 4 presented with synkinesia, dental agenesis, growth retardation, and myasthenia gravis; Patient 5 had SOD, hearing loss, and intellectual disability; and patient 7 was diagnosed with congenital ocular motor dyspraxia in childhood. In six cases, the variant was inherited from one parent. In all but two families (patients 3 and 5), the transmitting parent was asymptomatic, consistent with the incomplete penetrance previously described for *FGFR1* mutations [28]. For patient 7, parental DNA was not available for genetic analysis.

The remaining three patients carried heterozygous deleterious variants in three different genes: *CHD7*, *SOX9*, and *CTNND1* (Table 1). Patient 8, a female with a LP *CHD7* variant (p.Ala1107Val), exhibited anosmia and a complex phenotype including hearing loss, skeletal anomalies, synkinesia, mitral valve prolapse, atrioventricular nodal reentry tachycardia, astigmatism, choroidal nevus, vesicoureteral reflux, and unilateral renal hypoplasia. The variant was inherited from her mother, who had hearing loss. Her sister, who also carried the variant, presented with both CHH and CLP; patient 9, a female, harbored a de novo pathogenic *SOX9* variant (p.Pro170Arg). She exhibited anosmia and multiple anomalies, including ear malformations, growth retardation, hearing loss, intellectual and developmental disability (ID/DD), bicuspid aortic valve, synkinesia, strabismus, metatarsus varus, brachydactyly, and low-set ears; and Patient 10, a male, carried a LP splice-site variant in *CTNND1* (c.2809-1 G > C), inherited from his affected father, who had delayed puberty and CLP. The proband exhibited hypertelorism, double permanent teeth, hearing loss, unilateral cryptorchidism, and brachydactyly. He had four brothers: three with facial clefts who also carried the variant, and one with delayed puberty who did not carry the variant.

Overall, among the 10 index patients, only two had a normal sense of smell, while five presented with complex associated phenotypes—hearing loss being the most common, observed in four individuals.

### Probands with CHH without CLP harbor P/LP variants in CLP genes

**Fig. 1:**
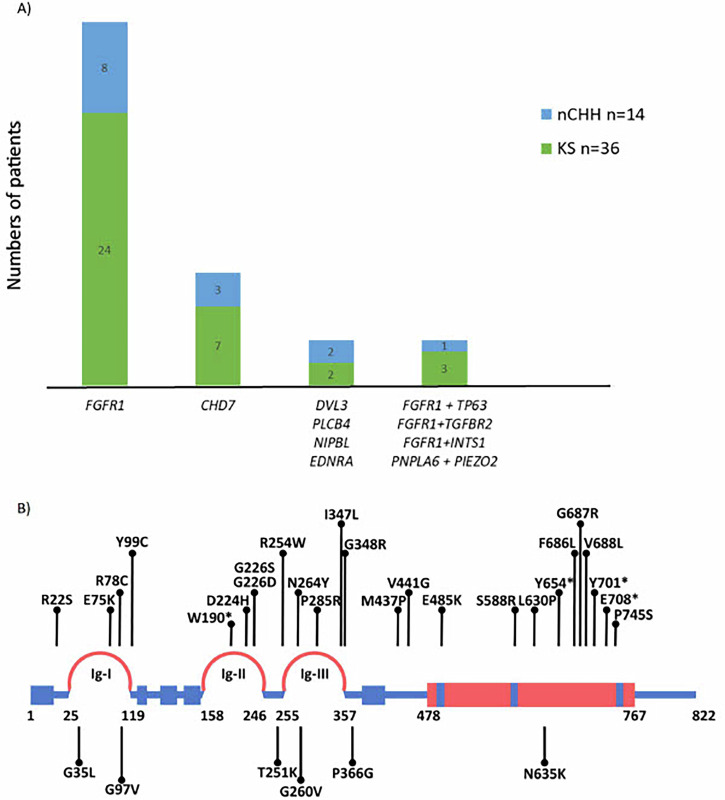
Likely pathogenic (LP) and pathogenic (P) variants in CLP-related genes among a cohort of 336 patients with CHH. **A** LP/P variants in CLP genes in 50 CHH patients without CLP. **B** 35 *FGFR1* mutations in 336 probands with CHH. *FGFR1* mutations in patients with CHH and CLP are depicted below.

Among the remaining 315 probands with CHH without CLP, 50 harbored P and LP variants in CLP-associated genes, representing 15.8% of the cohort (Fig. 1). Notably, four cases exhibited potential digenic inheritance involving both CLP-related and CHH-associated genes (Fig. 1).

### Probands with CHH without CLP harboring deleterious variants in overlapping genes for CHH and CLP

*FGFR1* and *CHD7* are individually implicated in both CHH and CLP. Among 315 patients with CHH without CLP, 32 patients (22 males) carried P or LP variants in FGFR1 (Supplementary Table S2). The identified *FGFR1* variants included a range of deleterious types: missense, nonsense, frameshift, and splice-site-altering mutations. Inheritance could be evaluated in five cases, and four variants were confirmed as de novo (Supplementary Table S2, patients 19, 24, 26, and 30).

Phenotypically, 27 of the 32 patients were anosmic or hyposmic (Fig. 1A and Supplementary Table S2). Several individuals exhibited complex associated anomalies: patient 33 had agenesis of the septum pellucidum, consistent with a diagnosis of SOD in the context of CHH; dental agenesis was observed in patients 39 and 48; patient 24 had an atrial septal defect; patient 29 exhibited growth retardation; patient 49 had a diagnosis of autism spectrum disorder; and patient 47 presented with ear anomalies, type 2 diabetes, enuresis, and genu valgum (Supplementary Table S2).

For *CHD7*, the major causative gene for CHARGE syndrome, we identified P/LP variants in 10 patients with CHH (7 males) (Supplementary Table S3). These included eight missense variants, one nonsense (stop-gain) variant, and one splice-site variant. KS was diagnosed in seven of these individuals, while the remaining three were classified as having normosmic CHH (nCHH). Three patients (patients 54, 58, and 59) met clinical criteria for CHARGE syndrome. Other associated phenotypes among *CHD7* variant carriers included hearing loss, SOD, and congenital cataracts.

### Probands with CHH without CLP harboring deleterious variants in genes exclusively associated with CLP

In our cohort of 315 CHH patients without cleft, we found 4 patients (1 female) with heterozygous deleterious variants in genes exclusively associated with CLP—*DLV3*, *PLCB4*, *NIPBL* and *EDNRA*. Three variants were frameshifts leading to a premature stop codon, and one was splice-site altering (Table 2). Two patients had KS, and 2 patients were normosmic. As associated phenotypes, patient 12 had obesity and gynecomastia, and patient 13 had Cornelia de Lange Syndrome. The inheritance pattern was possible to be established only in patient 13, de novo. Pathogenic variants in these genes were previously associated with genetic syndromes (Table 3). Notably, three of these syndromes have clinical features in common with CHH, such as cryptorchidism, micropenis and hypoplasia of the corpus callosum.

**Table 2:** Genetic and clinical data of patients with CHH without cleft lip and/or palate carrying variants in CLP genes.

Patient	Sex	Genes	Nucleotide or amino acid change	ACMG class	Olfaction	Neuroimaging	Associated phenotypes
11	M	*DVL3*	p.Ile109Metfs*50	LP	Anosmia	Normal OBs but shallow sulci	None
12	M	*PLCB4*	p.Ala532Cysfs*3	LP	Normal	Normal brain, pituitary, OBs and sulci	Obesity, gynecomastia
13	M	*NIPBL*	p.Pro2761Cysfs*4	LP	Hyposmia	Absent left OB, reduced right OB	CdLS
14	F	*EDNRA*	c.747 + 1 G > A^a^	LP	Normal	Normal pituitary	None

All variants were in heterozygous state. Inheritance was not able to be evaluated.

*M* male; *F* female; *ACMG* *class* American College of Medical Genetics classification; *LP* likely pathogenic; *OB* olfactory bulb; *CdLS* cornelia de Lange syndrome.

^a^Predicted to be splice altering using the Splice AI tool.

**Table 3:** Function and phenotypes of putative novel genes for CHH.

Gene	Type of inheritance	Function	Expression in HP	OMIM phenotype	Reproductive phenotype/ associated CHH phenotype
*DVL3*	AD	Involved in Wnt signaling pathway	Yes	Robinow syndrome	Cryptorchidism
*PLCB4*	AD/AR	Signal transduction via the PI cycle	Yes	Auriculocondylar syndrome 2 and 2B	Macropenis, macroorchidism, hydrocele, clitoral hypertrophy, hypoplasia of corpus callosum
*NIPBL*	AD	Member of the cohesin complex	Yes	CdLS1	Cryptorchidism, hypoplastic male genitalia
*EDNRA*	AD	Receptor for endothelin-1.	Yes	Mandibulofacial dysostosis with alopecia	–

*DVL3* disheveled segment polarity protein 3; *PLCB4* phospholipase C beta 4; *NIPBL* NIPBL cohesin loading factor; *EDNRA* endothelin receptor type A; *AD* autossomic dominant; *AR* autossomic ressessive; *PI* phosphatidylinositol; *CdLS1* cornelia de Lange syndrome 1.

### Probands with CHH without CLP harboring digenic P/LP variants

We identified four patients with CHH in our cohort who carried more than one deleterious variant in the CLP gene panel and/or CHH gene panel (Table 4), suggesting possible digenic or oligogenic inheritance. In three of these cases, *FGFR1* variants co-occurred with variants in *TP63*, *TGFBR2*, and *INTS1*, respectively. Patient 18 has compound heterozygous LP variants in *PNPLA6* and an additional deleterious variant in *PIEZO2*. Regarding associated phenotypes: patient 16 exhibited dental agenesis; patient 17 presented with oxycephaly (craniosynostosis of the sagittal and coronal sutures); and patient 18 showed evidence of an empty sella on neuroimaging, along with vermis hypoplasia and ataxia.

**Table 4:** Genetic and clinical data of patients with CHH with digenic LP or P variants.

Patient	Sex	Gene	Nucleotide or amino acid change	Inheritance	ACMG class	Olfaction	Neuroimaging	Associated phenotypes
15	M	*TP63* *FGFR1*	c.63-1 G > C p.Arg78Cys	Unknown	LP LP	Anosmia	Absent OB, normal pituitary	Bilateral cryptorchidism
16	M	*TGFBR2* *FGFR1*	p.Glu485Lys p.Ile347Leufs*11	Paternal Paternal	LP P	Anosmia	NA	Dental agenesis
17	F	*INTS1* *FGFR1*	p.Val206Alafs*4 p.Pro285Arg	Unknown	LP LP	Anosmia	NA	Oxycephaly
18	F	*PIEZO2* *PNPLA6* *PNPLA6*	c.330-2dup p.Gly1240Arg p.Gly1002Ser	Paternal Paternal Maternal	LP LP LP	Normal	Empty sella and vermis hypoplasia	Ataxia

*M* male; *F* female; *ACMG* *class*, American College of Medical Genetics classification; *LP* likely pathogenic; *P* pathogenic; *OB* olfactory bulb; *NA* not available.

All variants are in heterozygous state.

*TP63, FGFR1, TGFBR2* have autosomal dominant (AD) mode of inheritance (OMIM).

*INTS1* and *PNPLA6* have autosomal recessive (AR) mode of inheritance (OMIM).

*PIEZO2* have both AD and AR modes of inheritance (OMIM).

## Discussion

CHH presents with a wide spectrum of associated phenotypes, including well-defined syndromic forms, such as KS, SOD, and CHARGE syndrome [19]. This clinical heterogeneity reflects the shared embryological origins of the cranial placodes and the GnRH neuronal system, which contribute to both olfactory and neuroendocrine development [3, 19]. While midline craniofacial anomalies are reported in approximately 9% of patients with CHH [15], prior studies have not systematically distinguished between subtypes, such as cleft lip vs. cleft palate, bifid uvula, ectopic posterior pituitary, or midline brain anomalies, including agenesis of the corpus callosum and septum pellucidum.

In this study, we analyzed a large cohort of 336 individuals with CHH to investigate the frequency and spectrum of pathogenic variants in genes associated with CLP. Clinical features were thoroughly reviewed, and a curated CLP gene panel was applied to identify P or LP variants. “Overall, 60 patients with P/LP variants were identified in approximately 17.8% of the cohort, highlighting a significant genetic overlap between CHH and CLP.”

Among the 21 patients with CHH who presented with CLP (6%), 10 harbored P/LP variants in CLP-related genes, five of whom exhibited complex associated phenotypes. Notably, 8 of these 10 P/LP variants were in genes known to be implicated in both CHH and CLP. The most frequently implicated gene was *FGFR1* (*n* = 7), followed by *CHD7*, *SOX9*, and *CTNND1*. Both *FGFR1* and *CHD7* are also CHH-related genes. In the remaining 315 CHH patients without CLP, we identified 54 P/LP variants in CLP-related genes in 50 individuals, including four cases suggestive of digenic inheritance. Across the full cohort with and without CLP, *FGFR1* (*n* = 42) and *CHD7* (*n* = 11) emerged as the most affected genes, reinforcing their central role in the overlapping genetic architecture of CHH and CLP. In the 10 families, it is possible to observe two phenomena: incomplete penetrance and variable clinical expressivity, the latter manifested as constitutional delay of growth and puberty (CPHD). Both events have been described by our group and others [5, 7].

*FGFR1* is a pleiotropic gene involved in the development of GnRH neurons as well as in craniofacial and limb morphogenesis [34, 35]. This explains its association with diverse syndromes such as CHH, CLP, split-hand/foot malformation, and Hartsfield syndrome [36, 37]. Likewise, *CHD7*—the primary gene implicated in CHARGE syndrome—is known to contribute to both CHH and CLP phenotypes [10, 38]. Murine models have demonstrated that *Chd7* plays a key developmental role in GnRH neuron migration and olfactory system formation, further supporting its relevance in the shared pathogenesis of these conditions [39, 40]. In two patients with CHH and CLP, deleterious variants were found in *SOX9* and *CTNND1*. Although both are clear linked with the CLP, this is not the case for CHH. Both genes are involved in complex phenotypes. Of note, *SOX9* in implicated in skeletal dysplasia, hearing loss and absence of the olfactory bulbs, resembling patient 9 [41]. Regarding *CTNND1,* patients can manifest tooth abnormalities as observed in our patient [42]. The pathophysiological mechanism underlying CHH associated with these genes remains to be established.

Of particular interest, we identified four patients with CHH without CLP who carried P/LP variants in genes previously associated exclusively with CLP, including *DLV3*, *PLCB4*, *NIPBL*, and *EDNRA*. These genes are known to underlie various congenital anomaly syndromes, some of which include reproductive features, such as micropenis or cryptorchidism, or neurodevelopmental anomalies such as corpus callosum hypoplasia—findings that partially overlap with the CHH phenotype [43–45]. One exception is *EDNRA*, where pathogenic variants cause mandibulofacial dysostosis with alopecia, a condition not typically linked to reproductive dysfunction [40, 46]. The contribution of these genes to the pathophysiology of CHH remains unclear, and functional studies are needed to determine whether they disrupt GnRH neuron development, pituitary function, or downstream hormonal regulation. Integrated approaches using transcriptomics and model organisms may help elucidate novel developmental links between craniofacial and reproductive systems.

Additionally, our observation of patients harboring multiple deleterious variants—including combinations of CHH- and CLP-related genes—supports an oligogenic model of inheritance. This may help explain the clinical variability.

Our findings underscore the importance of heightened clinical awareness among craniofacial teams, pediatricians, and geneticists regarding the potential reproductive and neuroendocrine sequelae in patients with CLP—especially those carrying variants in *FGFR1* or *CHD7*. Because CLP is typically diagnosed at birth, clinical attention is often directed toward surgical and structural concerns, while endocrine abnormalities such as delayed or absent puberty may remain undetected until adolescence or adulthood. Early genetic screening of CLP patients for mutations in overlapping genes (i.e., *FGFR1* and *CHD7*) may enable timely identification of individuals at risk for CHH, facilitating early endocrine evaluation and intervention. Moreover, future studies are warranted to assess the true prevalence of CHH within CLP cohorts, particularly in those with relevant genetic variants, to further define at-risk populations and optimize clinical management.

In conclusion, this study highlights a significant genetic and phenotypic overlap between CHH and CLP, driven in part by shared developmental pathways and pleiotropic genes, such as *FGFR1* and *CHD7*. The identification of pathogenic variants in CLP-related genes—both in patients with CHH with and without craniofacial anomalies—supports a broader view of CHH as a developmental disorder with variable expressivity and potential oligogenic inheritance. These findings emphasize the need for integrated clinical and genetic screening strategies in both CHH and CLP populations to enable earlier diagnosis, improve genetic counseling, and guide personalized care.

## Supplementary information

Supplementary Table S1

Supplementary Table S2

Supplementary Table S3

## Data Availability

The data generated and analyzed during this study can be found within the published article and its supplementary files. Additional data is available from the corresponding author on reasonable request.
