# Supplementary material for: Pleiotropic genes linking congenital hypogonadotropic hypogonadism and cleft lip/palate: evidence from a genomic CHH cohort study
Source: Eur J Hum Genet. 2026 Jan 14;34(3):340–7. doi: 10.1038/s41431-025-02005-6 (PMC12963409; doi:10.1038/s41431-025-02005-6)
Supplement: Supplementary file 1 — Supplementary Table S1 [file 41431_2025_2005_MOESM1_ESM.docx]

**Supplementary Table S1 – Genetic panels – FGFR1 and CHD7 are present in both panels.**

| Genes for CLP – panel A | Genes for CHH – panel B |
| --- | --- |
| ACTB | ANOS1 |
| ACTG1 | CHD7 |
| ALX1 | CPE |
| AMER1 | DMXL2 |
| AMOTL1 | DUSP6 |
| ANKRD11 | FEZF1 |
| ARHGAP29 | FGF17 |
| ARHGAP31 | FGF8 |
| ASXL1 | FGFR1 |
| B3GLCT | FLRT3 |
| BCOR | FSHB |
| BMP2 | GNRH1 |
| C2CD3 | GNRHR |
| C5orf42 | HS6ST1 |
| CC2D2A | IL17RD |
| CDH1 | KISS1 |
| CDKN1C | KISS1R |
| CHD7 | LEP |
| CHRNG | LEPR |
| CHST14 | LHB |
| CNTNAP1 | NDNF |
| COL11A1 | NHLH2 |
| COL11A2 | NR0B1 |
| COL2A1 | NSMF |
| COL9A1 | PCSK1 |
| COLEC10 | PNPLA6 |
| COLEC11 | POLR3A |
| CTCF | POLR3B |
| CTNND1 | PRDM13 |
| DHCR7 | PROK2 |
| DHODH | PROKR2 |
| DLL4 | RNF216 |
| DOCK6 | SEMA3A |
| DVL1 | SMCHD1 |
| DVL3 | SOX10 |
| DYNC2H1 | SOX11 |
| DYNC2LI1 | SPRY4 |
| EBP | TAC3 |
| EDNRA | TACR3 |
| EFNB1 | TCF12 |
| EFTUD2 | WDR11 |
| EIF2S3 |  |
| EIF4A3 |  |
| EOGT |  |
| EPG5 |  |
| ESCO2 |  |
| EYA1 |  |
| FAM20C |  |
| FGD1 |  |
| Genes for CLP – panel A |  |
| FGFR1 |  |
| FGFR2 |  |
| FLNA |  |
| FLNB |  |
| FOXC2 |  |
| FRAS1 |  |
| GDF11 |  |
| GJA1 |  |
| GLI2 |  |
| GLI3 |  |
| GPC3 |  |
| GRHL3 |  |
| HDAC8 |  |
| HNRNPK |  |
| HYAL2 |  |
| HYLS1 |  |
| ICK |  |
| IFT140 |  |
| IFT172 |  |
| IFT80 |  |
| IMPAD1 |  |
| INTS1 |  |
| IRF6 |  |
| KAT6A |  |
| KAT6B |  |
| KCNJ2 |  |
| KDM6A |  |
| KIAA0586 |  |
| KIF1BP |  |
| KIF7 |  |
| KMT2D |  |
| MAP3K7 |  |
| MAPRE2 |  |
| MASP1 |  |
| MBTPS2 |  |
| MED12 |  |
| MED25 |  |
| MEIS2 |  |
| MID1 |  |
| MKS1 |  |
| MSX1 |  |
| MYMK |  |
| NECTIN1 |  |
| NEDD4L |  |
| NEK1 |  |
| NIPBL |  |
| NOTCH1 |  |
| OFD1 |  |
| PAX3 |  |
| PGAP3 |  |
| PGM1 |  |
| Genes for CLP – panel A |  |
| PHF8 |  |
| PIEZO2 |  |
| PIGN |  |
| PIGV |  |
| PLCB4 |  |
| POLR1B |  |
| POLR1C |  |
| POLR1D |  |
| PORCN |  |
| PTCH1 |  |
| RAD21 |  |
| RBM10 |  |
| ROR2 |  |
| RPL5 |  |
| RPS26 |  |
| SALL4 |  |
| SATB2 |  |
| SCARF2 |  |
| SF3B2 |  |
| SF3B4 |  |
| SHH |  |
| SIX1 |  |
| SIX3 |  |
| SIX5 |  |
| SKI |  |
| SLC26A2 |  |
| SMAD3 |  |
| SMAD4 |  |
| SMARCA4 |  |
| SMC1A |  |
| SMC3 |  |
| SMS |  |
| SNRPB |  |
| SON |  |
| SOX9 |  |
| SPECC1L |  |
| STAG2 |  |
| STAMBP |  |
| TBX1 |  |
| TBX22 |  |
| TCOF1 |  |
| TCTN3 |  |
| TELO2 |  |
| TFAP2A |  |
| TGDS |  |
| TGFB3 |  |
| TGFBR1 |  |
| TGFBR2 |  |
| TMCO1 |  |
| TP63 |  |
| TRAPPC9 |  |
| Genes for CLP – panel A |  |
| TRIM37 |  |
| TRRAP |  |
| TUBB |  |
| TXNL4A |  |
| USP9X |  |
| WNT5A |  |
| XYLT1 |  |
| ZC4H2 |  |
| ZEB2 |  |
| ZIC2 |  |
| ZIC3 |  |
| ZSWIM6 |  |
