# Supplementary material for: Pleiotropic genes linking congenital hypogonadotropic hypogonadism and cleft lip/palate: evidence from a genomic CHH cohort study
Source: Eur J Hum Genet. 2026 Jan 14;34(3):340–7. doi: 10.1038/s41431-025-02005-6 (PMC12963409; doi:10.1038/s41431-025-02005-6)
Supplement: Supplementary file 2 — Supplementary Table S2 [file 41431_2025_2005_MOESM2_ESM.docx]

**Supplementary Table S2 – Patients with CHH without CLP with Pathogenic and Likely Pathogenic heterozygous variants in *FGFR1* (NM_023110).**

| Patient | Sex | Nucleotide  Change | Amino Acid  Change | Inheritance | ACMG  class | Diagnosis | Neuroimaging | Associated  Phenotypes |
| --- | --- | --- | --- | --- | --- | --- | --- | --- |
| 19 | F | c.2058del | p.Phe686Leufs*28 | *de novo* | LP | KS | na | - |
| 20 | M | c.670G>C | p.Asp224His | na | LP | KS | na | - |
| 21 | M | c.2122G>T | p.Glu708* | na | P | KS | na | - |
| 22 | M | c.92-1G>T | - | na | LP | KS | na | - |
| 23 | F | c.1430+1del | - | na | LP | KS | na | - |
| 24 | M | c.1889T>C | p.Leu630Pro | *de novo* | LP | nCHH | na | Atrial septal defect |
| 25 | F | c.1756_1763dup | p.Ser588Argfs*47 | na | LP | CHH | na | - |
| 26 | F | c.1042G>A | p.Gly348Arg | *de novo* | P | KS | Olfactory sulci, bulbs and  tracts severely hypoplastic |  |
| 27 | F | c.296A>G | p.Tyr99Cys | na | P | KS | na | - |
| 28 | M | c.1322_1334del | p.Val441Glyfs*13 | na | LP | KS | na | - |
| 29 | M | c.1042G>A | p.Gly348Arg | na | P | KS | Agenesis of olfactory bulbs | Growth retardation |
| 30 | M | c.2062G>T | p.Val688Leu | *de novo* | LP | KS | na | - |
| 31 | M | c.1961dup | p.Tyr654* | na | LP | KS | na | - |
| 32 | M | c.570G>A | p.Trp190* | na | LP | KS | na | - |
| 33 | M | c.622-1G>T | - | na | LP | nCHH | Agenesis of septum pellucidum | Septo-optic dysplasia |
| 34 | M | c.2233C>T | p.Pro745Ser | na | LP | KS | na | - |
| 35 | M | c.1977+1G>A | - | na | P | KS | na | - |
| 36 | F | c.296A>G | p.Tyr99Cys | na | P | KS | na | - |
| 37 | M | c.790A>T | p.Asn264Tyr | na | LP | KS | na | - |
| 38 | M | c.1306_1307dup | p.Met437Profs*2 | na | LP | nCHH | na | - |
| 39 | F | c.1042G>A | p.Gly348Arg | na | P | KS | na | Tooth Agenesis |
| 40 | M | c.66del | p.Arg22Serfs*81 | na | LP | nCHH | Normal pituitary | - |
| 41 | M | c.1977+1G>A | - | na | P | nCHH | na | - |
| 42 | F | c.677G>A | p.Gly226Asp | na | LP | KS | na | - |
| 43 | M | c.2059G>A | p.Gly687Arg | na | P | KS | na | - |
| 44 | F | c.676G>A | p.Gly226Ser | na | LP | nCHH | na | - |
| 45 | M | c.223_224del | p.Glu75Lysfs*35 | na | P | KS | Normal olfactory bulbs | - |
| 46 | M | c.2103C>G | p.Tyr701* | na | LP | KS | Agenesis of olfactory bulbs | - |
| 47 | F | c.760C>T | p.Arg254Trp | na | LP | nCHH | Normal pituitary | Ear anomalies, Type 2 Diabetes, enuresis, genu valgum |
| 48 | M | c.1825C>T | p.Arg609* | na | P | KS | Normal pituitary | Tooth agenesis, hyperlaxity |
| 49 | M | c.232C>T | p.Arg78Cys | na | LP | KS | na | Autism spectrum disorder |
| 50 | M | c.962_963del | p.Lys321Argfs*13 | na | P | KS | na | - |

F, female; M, male; na, not available; ACMG class, American College of Medical Genetics classification; LP, likely pathogenic; P, pathogenic; KS, Kallmann syndrome; nCHH, normosmic CHH; CHH, congenital hypogonadotropic hypogonadism.
