# Supplementary material for: Pleiotropic genes linking congenital hypogonadotropic hypogonadism and cleft lip/palate: evidence from a genomic CHH cohort study
Source: Eur J Hum Genet. 2026 Jan 14;34(3):340–7. doi: 10.1038/s41431-025-02005-6 (PMC12963409; doi:10.1038/s41431-025-02005-6)
Supplement: Supplementary file 3 — Supplementary Table S3 [file 41431_2025_2005_MOESM3_ESM.docx]

**Supplementary Table S3 – Deleterious variants in *CHD7* (NM_017780) in patients with CHH without CLP.**

| **Patient** | **Sex** | **Nucleotide**  **Change** | **Amino acid**  **Change** | **ACMG**  **Class** | **Diagnosis** | **Neuroimaging** | **Associated Phenotypes** |
| --- | --- | --- | --- | --- | --- | --- | --- |
| 51 | M | c.3056T>G | p.Phe1019Cys | LP | KS | Absent OB,  normal pituitary | Marfanoid habitus, High-arched palate, bilateral orchiopexy |
| 52 | F | c.6193C>G | p.Arg2065Gly | LP | KS | na | Hearing loss, Left sensorineural deafness |
| 53 | M | c.5945G>A | p.Gly1982Glu | LP | KS | na | Hearing loss, Optic nerve hypoplasia (SOD), bilateral cryptorchidism |
| 54 | M | c.2966G>A | p.Cys989Tyr | LP | KS | Absent OB,  small pituitary | CHARGE syndrome, coloboma, ear anomalies, growth retardation, hearing loss, convergent strabismus, facial palsy. |
| 55 | M | c.2613+5G>A | - | LP | nCHH | na | No |
| 56 | M | c.3884T>G | p.Ile1295Ser | LP | KS | na | No |
| 57 | M | c.6955C>T | p.Arg2319Cys | P | nCHH | na | No |
| 58 | M | c.3106C>T | p.Gly260Val | P | nCHH | Normal brain MRI | CHARGE syndrome, coloboma, choanal atresia, keratocone, ID/DD, short stature, obesity, hearing loss, cup ears and clinodactyly. |
| 59 | F | c.1777C>T | p.Gln593* | LP | KS | Normal OB,  normal pituitary | CHARGE syndrome Choanal atresia, Hearing loss, hypoacusis conductive, atrial and ventricular septum defect, ductus arteriosus persistency, coarctation aortae |
| 60 | F | c.6955C>T | p.Arg2319Cys | P | KS | na | Congenital cataracta and strabismic amblyopia. |

M, male; F, female; ACMG class, American College of Medical Genetics classification; LP, likely pathogenic; P, pathogenic; KS, Kallmann syndrome; nCHH, normosmic CHH; OB, olfactory bulb; na, not available; MRI, magnetic resonance imaging; SOD, septo-optic dysplasia; ID/DD, intellectual deficiency/developmental delay.
